# Lipase-catalyzed biodiesel production and quality with *Jatropha curcas oil*: exploring its potential for Central America

**DOI:** 10.1186/s13036-015-0009-9

**Published:** 2015-07-24

**Authors:** Francisco Bueso, Luis Moreno, Mathew Cedeño, Karla Manzanarez

**Affiliations:** Department of Food Science and Technology, EAP Zamorano University, P.O. Box 93, Tegucigalpa, Honduras

**Keywords:** Biodiesel, Crude oil, *Jatropha curcas*, Transesterification, Soluble lipase, ASTM D675

## Abstract

**Background:**

Extensive native *Jatropha curcas L*. (Jatropha) crop areas have been planted in Central America marginal lands since 2008 as a non-edible prospective feedstock alternative to high-value, edible palm oil. Jatropha biodiesel is currently exclusively produced in the region at commercial scale utilizing alkaline catalysts. Recently, a free, soluble *Thermomyces lanuginosus* (TL) 1,3 specific lipase has shown promise as biocatalyst, reportedly yielding up to 96 % ASTM D6751 compliant biodiesel after 24 h transesterification of soybean, canola oils and other feedstocks. Biodiesel conversion rate and quality of enzymatically catalyzed transesterification of Jatropha oil was evaluated. Two lipases: free, soluble TL and immobilized *Candida antarctica* (CA) catalyzed methanolic transesterification of crude Jatropha and refined palm oil.

**Results:**

Jatropha yields were similar to palm biodiesel with NaOH as catalyst. After 24 h transesterification, Jatropha (81 %) and palm oil (86 %) biodiesel yields with TL as catalyst were significantly higher than CA (<70 %) but inferior to NaOH (>90 %). Enzymatic catalysts (TL and CA) produced Jatropha biodiesel with optimum flow properties but did not complied with ASTM D6751 stability parameters (free fatty acid content and oil stability index).

**Conclusions:**

Biodiesel production with filtered, degummed, low FFA Jatropha oil using a free liquid lipase (TL) as catalyst showed higher yielding potential than immobilized CA lipase as substitute of RBD palm oil with alkaline catalyst. However, Jatropha enzymatic biodiesel yield and stability were inferior to alkaline catalyzed biodiesel and not in compliance with international quality standards. Lower quality due to incomplete alcoholysis and esterification, potential added costs due to need of more than 24 h to achieve comparable biodiesel yields and extra post-transesterification refining reactions are among the remaining drawbacks for the environmentally friendlier enzymatic catalysis of crude Jatropha oil to become an economically viable alternative to chemical catalysis.

## Background

Biodiesel can be produced with a variety of feedstock including refined bleached deodorized (RBD) edible vegetable oils, animal fats and waste cooking oils. The choice of feedstock depends mainly on geographical distribution [1] and price, which might amount up to 80 % of production costs [2]. Palm (*Elaeis guineensis*) has been the preferred oil crop for industrial biodiesel production in Central America due to its extensive cultivation in the region (specially in Honduras) and high (3.5–5 t/ha) oil yields (2). Extensive native *Jatropha curcas L*. (Jatropha) crop areas have been planted in marginal lands since 2008 in the region as a non-edible prospective feedstock alternative to high-value, edible palm oil.

Biodiesel is currently mostly produced at commercial scale utilizing alkali, mainly sodium hydroxide [1–5]. Process limitations such as presence of soap-forming free fatty acids (FFA) in quantities above 0.5 % are considered drawbacks of chemical biodiesel [1, 2]. Furthermore, the by-products and waste water from the process act as potential environment pollutants [5]. An acid-catalyzed pre-treatment becomes necessary prior to methanolic transesterification of crude Jatropha oil, which normally contains >15 % FFA in order to reach 90–99 % biodiesel yields [3].

The use of non-specific and 1,3-specific lipases that can catalyze both esterification of FFA and transesterification of triacylglycerols (TGs) in the oil and yield cleaner by-products as an alternative to harmful and hard to manage acid or alkali catalysts has been extensively documented [1, 2, 5, 6].

Immobilized, non-specific *Candida antarctica* (CA) lipase B (Novozym 435) has been the most commonly investigated enzymatic catalyst for Jatropha biodiesel production [1, 2, 7]. However, biodiesel yields (72–80 %) have been inferior compared to basic catalysts for methanolic transesterification at 10–30 % *w*/*w* even after 90 h [1, 2, 8, 9]. Recently a free, soluble *Thermomyces lanuginosus* (TL) 1,3 specific lipase at 0.75 % *w*/*w*, 1.5:1 methanol:oil ratio and 2 % added water at 35 °C has shown promise as biocatalyst, reportedly yielding up to 96 % fatty acid methyl esters (FAME) after 24 h transesterification of soybean, canola oils and other feedstocks [3, 10–12].

Physicochemical properties of biodiesel should meet the quality requirements that are applicable to petrodiesel [2]. The US standard for biodiesel is stipulated in the American Society for Testing Materials (ASTM) D6751 [13]. Fuel properties of Jatropha biodiesel are considered as good as petro-diesel with better cooling properties than palm oil [14].

Two commercial enzymatic biodiesel plants with production capacities over 1 million gallons per year already operate in Florida and North Carolina (United States of America) claiming economic feasibility and compliance with ASTM D6751 standard using 1,3 specific lipases to catalyze transesterification of soybean oil and other feedstocks [11, 15].

Compliance of alkali-catalyzed Jatropha biodiesel with ASTM D6751 has been well documented [1, 14]. However, lipase-catalyzed Jatropha biodiesel compliance has been reported scarcely, and lack stability parameters [2]. To fill this gap in the literature, the potential of lipase-catalyzed biodiesel as an alternative to conventional alkaline transesterification was evaluated with Jatropha oil in comparison to palm oil. For this purpose, the catalytic performance of a free, liquid TL lipase in terms of biodiesel yield and quality was evaluated vs. immobilized CA.

## Results and discussion

### Biodiesel yields

Jatropha oil biodiesel yields were similar to palm oil with basic catalyst (Table 1). After 24 h transesterification, Jatropha and palm oil biodiesel yields with TL as catalyst were significantly higher than CA but inferior to NaOH (Table 1). Biodiesel at yields 94–99 % is conventionally manufactured from vegetable oils using sodium or potassium methoxyde at concentrations of 0.5–1 wt.% to complete transesterification of lipids in several hours [1]. Chemical transesterification of Jatropha oil has been reported to yield over 90 % FAME in 1–1.5 h as long as FFA is below 1 % [2], as was the case in this study. Bacterial and fungal lipases have been reported to esterify FFA in partially refined and used oils to yield 90–99 % FAME in 24–90 h and make the process more economically viable [1, 4–6, 16, 17]. Liquid lipases can be produced and sold at a much lower price than immobilized lipases [10, 11]. Liquid TL lipase has shown promising biodiesel yields (92–96 %) with sunflower and soybean oil [1, 3, 5, 10]. It was not the case with degummed Jatropha (81 %) and RBD palm oils (86 %) in this study (Table 1).

**Table 1:** Biodiesel yield (%) from palm and Jatropha oil after 24 h transesterification with enzymatic catalysts

Catalyst	Oil	Biodiesel yield
		% ± S.D.
NaOH	Jatropha	90.0 ± 2.6^a^
	Palm	92.3 ± 1.5^a^
TL	Jatropha	80.7 ± 2.5^b^
	Palm	85.6 ± 4.0^b^
CA	Jatropha	66.8 ± 0.5^c^
	Palm	61.6 ± 0.9^c^
C.V. (%)		2.8

Data are from transesterified oils (Jatropha and palm) with alkaline (NaOH) and enzymatic (TL and CA) catalysts. Means with different superscript letters (a, b, c) on the same column are significantly different (LSD test, *P* < 0.05). % *C.V*. percent coefficient of variation

Yields with immobilized CA lipase as catalyst were significantly inferior than TL and NaOH for palm and Jatropha oil after 24 h. These results are in line with those obtained when CA was used as catalyst and methanol as acyl acceptor for Jatropha biodiesel production even after 90 h of reaction time [2, 3, 9]. Immobilization of CA in acrylic resin has been thought to confer more effective activity than free, soluble lipases such as TL due to more exposition of active sites [7]. However, under reaction conditions of this study (using methanol as acyl acceptor, particularly) free, soluble TL was able to produce biodiesel from vegetable oils with significantly higher efficiency than immobilized CA [3].

### Biodiesel quality

Alkaline (NaOH) and enzymatic catalysts (TL and CA) produced palm and Jatropha biodiesel with optimum viscosity, cloud point and cetane number according to ASTM D6751 standard [13] at levels similar to previous studies [1, 2, 18] (Table 2). Jatropha biodiesel lower viscosity and cloud point than palm biodiesel (due to lower saturated FAME content) have better tank to engine flow properties in temperate climates [1, 2] or during cooler months (November–February) in Central America.

**Table 2:** Flow properties and stability of biodiesel from palm and Jatropha oil

Catalyst	Oil	FFA	Viscosity	Cloud point	OSI	Cetane number
		mg KOH/g ± S.D.	(mm^2^/s) ± S.D.	(°C) ± S.D.	h ± S.D.	CN ± S.D.
NaOH	Jatropha	0.1 ± 0.1^d^	2.7 ± 0.04^d^	3.8 ± 0.18^b^	4.1 ± 0.1^b^	54.8 ± 0.3^b^
	Palm	0.1 ± 0.1^d^	4.7 ± 0.07^b^	14.3 ± 1.0^a^	10.5 ± 0.1^a^	63.3 ± 0.5^a^
TL	Jatropha	14.7 ± 0.4^b^	3.0 ± 0.04^c^	4.3 ± 0.29^b^	0.5 ± 0.4^d^	54.8 ± 0.1^b^
	Palm	10.8 ± 0.4^c^	5.1 ± 0.07^a^	14.8 ± 1.0^a^	3.9 ± 0.1^c^	65.6 ± 1.3^a^
CA	Jatropha	16.1 ± 1.2^a^	3.0 ± 0.04^c^	4.1 ± 0.30^b^	0.4 ± 0.5^d^	56.0 ± 0.4^b^
	Palm	13.9 ± 0.2^b^	4.9 ± 0.07^ab^	14.7 ± 1.0^a^	4.7 ± 0.1^b^	65.6 ± 0.1^a^
ASTM D6751		0.5 Maximum	1.9–6	Report	3 Minimum	47 Minimum
C.V. (%)		5.5	1.4	6.9	7.8	4.5

Data are from biodiesel quality parameters of transesterified oils (Jatropha and palm) with alkaline (NaOH) and enzymatic (TL and CA) catalysts compared to ASTM D6751 limits. Means with different superscript letters (a, b, c, d) on the same column are significantly different (LSD test, *P* < 0.05). % *C.V*. percent coefficient of variation

**Fig. 1:**
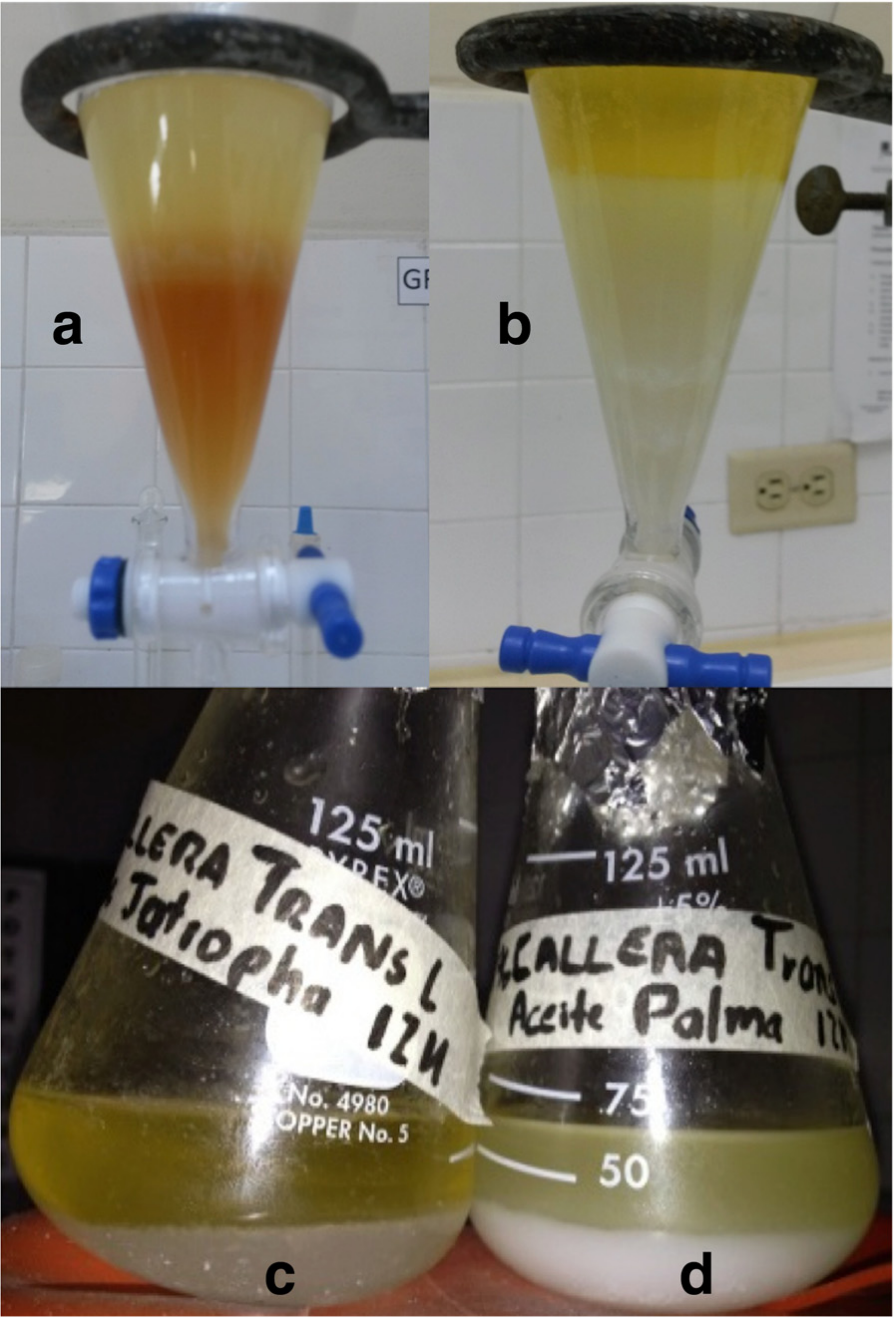
Enzymatic biodiesel produced with Jatropha and palm oil. **a** The alkaline-catalyzed biodiesel produces a reddish-brown glycerol phase. **b** Enzymatic biodiesel catalyzed by CA produced a cleaner (*white*) glycerol phase. **c** Jatropha biodiesel catalyzed by TL. **d** Palm biodiesel produced with TL enzymatic catalyst. One advantage of enzymatic biodiesel over alkaline catalyzed biodiesel is a cleaner, higher-quality glycerine by-product

Biodiesel from palm and Jatropha oil produced with enzymatic catalysts (Fig. 1) had higher FFA content than the maximum allowed by ASTM D6751, while biodiesel catalyzed by NaOH (Fig. 1a) complied with the standard (Table 2). Biodiesel produced with CA (Fig. 1b) as catalyst had significantly higher FFA than biodiesel catalyzed by TL (Fig. 1c, d). Jatropha biodiesel (Fig. 1c) had higher FFA content than palm biodiesel (Fig. 1d) when enzymatic catalysts were used (Table 2). FFA content of palm and Jatropha oils was <0.5 % before transesterification. Therefore, the high FFA content in palm and Jatropha biodiesel produced with TL (11–15 %) and especially CA (14–16 %) lipases suggest both enzymes were able to hydrolyze triacylglycerols into FFA, but could not completely esterify them with methanol into FAME within 24 h under conditions of this study (Table 2).

Enzymatic (TL and CA) Jatropha biodiesel did not comply with minimum ASTM D6751 stability (OSI) parameter, while enzymatic palm biodiesel did. Higher content of residual FFA (Table 2) and lower content of saturated FAME (Table 3) in Jatropha compared to palm biodiesel caused lower oxidation stability. Jatropha biodiesel has been previously reported to comply with physicochemical and stability parameters of ASTM D6751 standard when alkaline catalyst was used [2]. Enzymatic Jatropha biodiesel has met physicochemical parameters of the standard, although compliance with acid value and stability parameters has not been previously reported [1]. Compliance of enzymatic Jatropha biodiesel with ASTM D6751 purity and stability parameters could be achieved by additional post-transesterification reaction steps. Previous studies have accomplished FFA removal by neutralization [11] or resin/ion-exchange [15] with other feedstocks, albeit potential cost increases compared to using alkaline and/or acid catalysts in Central America. Increasing reaction times above 24 h or enzyme concentration with TL has not produced significant yield increases [12]. Triacylglycerol hydrolysis to FFA (TL) followed by esterification to methanol (CA) and post-transesterification FFA removal has reportedly produced ASTM D6751 compliant biodiesel [11] with other various feedstocks.

**Table 3:** FAME profile of biodiesel from palm and Jatropha oil

FAME	NaOH		TL		CA	
	Palm % ± S.D.	Jatropha % ± S.D.	Palm % ± S.D.	Jatropha % ± S.D.	Palm % ± S.D.	Jatropha % ± S.D.
16:0	40.9 ± 1.7^b^	16.1 ± 0.7^d^	52.0 ± 4.2^a^	20.2 ± 0.0^c^	49.8 + 0.1^a^	18.2 + 1.0^d^
18:0	4.7 ± 0.1^d^	6.4 ± 0.1^b^	4.3 ± 0.9^d^	8.2 ± 0.2^a^	5.6 + 0.1^c^	8.8 + 0.2^a^
16:1 cis-9	0.7 ± 0.3^b^	1.0 ± 0.3^a^	0.8 ± 0.1^b^	0.0 ± 0.0^c^	0.1 + 0.0^c^	0.0 + 0.0^c^
18:1n9c cis-9	41.7 ± 1.2^a^	43.9 ± 0.4^a^	33.3 ± 2.3^c^	37.9 ± 0.1^b^	34.9 + 0.1^c^	40.2 + 0.5^b^
18:2n6 cis-9, 12	8.6 ± 0.2^c^	31.1 ± 0.0^ab^	6.3 ± 0.3^c^	33.1 ± 0.1^a^	6.7 + 0.1^c^	30.1 + 0.9^b^
∑ AC. Saturated	48.3 ± 1.7^b^	23.7 ± 1.0^d^	59.0 ± 2.9^a^	28.7 ± 0.2^c^	57.9 + 0.1^a^	28.8 + 1.1^c^
∑ AC. Monounsaturated	42.5 ± 1.5^ab^	44.9 ± 0.2^a^	34.1 ± 2.4^d^	38.0 ± 0.1^c^	35.4 + 0.1^cd^	40.4 + 0.5^bc^
∑ AC. Polyunsaturated	9.2 ± 0.2^c^	31.3 ± 1.1^ab^	6.9 ± 0.6^d^	33.4 ± 0.1^a^	6.7 + 0.1^d^	30.7 + 0.9^b^

Data are from biodiesel FAME profile obtained by GC-FID of transesterified oils (Jatropha and palm) with alkaline (NaOH) and enzymatic (TL and CA) catalysts. *S.D*. standard deviation. Listed fatty acid methyl esters (FAME) are: hexadecanoic (16:0), octadecanoic (18:0), cis-9 hexadecenoic (16:1 cis-9), cis-9 octadecenoic (18:1n9c cis-9) and cis-9, 12 octadecadienoic (18:2n6 cis-9, 12). Means with different superscript letters (a, b, c, d) on the same horizontal line are significantly different (LSD test, *P* < 0.05)

**Fig. 2:**
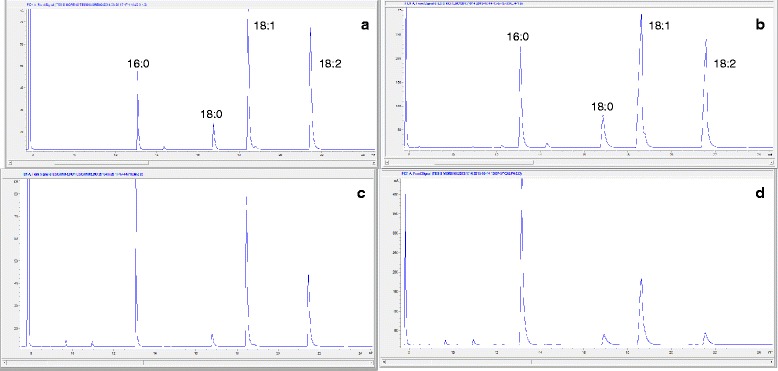
GC-FID FAME profile of enzymatic and alkali-catalyzed Jatropha and Palm biodiesel. **a** Chromatogram of Jatropha biodiesel catalyzed with NaOH. Fatty acid methyl esters (FAME) are: hexadecanoic (16:0), octadecanoic (18:0), cis-9 octadecenoic (18:1n9c cis-9) and cis-9, 12 octadecadienoic (18:2n6 cis-9, 12) **b** TL-catalyzed Jatropha biodiesel chromatogram. **c** Chromatogram of palm biodiesel catalyzed with NaOH. **d** Chromatogram of palm biodiesel produced with TL enzymatic catalyst

FAME profile of Jatropha (Fig. 2a) and palm biodiesel (Fig. 2c) produced with NaOH as catalyst were similar to reported fatty acid profiles of palm [19] and Jatropha [20] oils. In contrast, FAME profile of enzymatic biodiesel from Jatropha (Fig. 2b) and palm (Fig. 2d) oils were significantly different compared to biodiesel catalyzed by NaOH (Table 3). Saturated FAME (16:0 and 18:0) increased while unsaturated (18:1 cis-9) decreased. An increase in saturated FAME such as palmitic (16:0) and stearic (18:0) coupled with a decrease in unsaturated linolenic FAME (18:2) have been associated with increased cetane number in biodiesel produced from palm oil [21]. Changes in FAME profile of enzymatically-produced biodiesel did not significantly change cetane number compared to NaOH-catalyzed biodiesel (Tables 2, 3).

Transesterification of fatty acids from palm and Jatropha oil by CA and TL to biodiesel followed a similar pattern (Table 3). Unlike non-specific CA, TL express selectivity for the 1 and 3 positions in triacylglycerols, which means there might be an accumulation of 2-monoacylglycerols [9]. TL catalyst produced palm and Jatropha biodiesel yields over 66 % (Table 1), which would be the theoretical maximum. This is possible due to acyl migration in mono and diacylglycerols produced from triacylglycerol hydrolysis from position sn-2 to position sn-1 or sn-3 [9, 22–24]. Oleic and linoleic acid are mostly located on position sn-2 in palm oil triacylglycerols [19, 25], while in Jatropha oil the most common fatty acid found in position sn-2 is also oleic acid [26]. This suggests incomplete acyl migration from position sn-2 to sn-1,3 and/or incomplete transesterification of oleic acid occurred when TL lipase was used as catalysts of palm and Jatropha oil biodiesel production for 24 h. Up to 10 % monooleate has been found even after 48 h of CA and TL transesterification catalysis of vegetable oil [9] allowing for a 90 % maximum biodiesel yield.

Accumulation of non-hydrolized triacylglycerols, monooleate and other transesterification byproducts in addition to high FFA in the FAME phase may have caused the reduction in stability of biodiesel, especially Jatropha. Use of acyl migration additives [9, 24] and a combination of CA and TL as catalysts [9] to transesterify corn oil into biodiesel have yielded 90–94 % with less residual monooleate, although no compliance with quality standard has been reported.

## Conclusions

Biodiesel production with filtered, degummed, low FFA Jatropha oil using a free liquid lipase (TL) as catalyst and methanol as acyl acceptor showed higher yielding potential than immobilized CA lipase as substitute of RBD palm oil with alkaline catalyst. However, Jatropha enzymatic biodiesel yield and stability was inferior to alkaline catalyzed biodiesel and not in compliance with international quality standards. Lower quality–due to incomplete alcoholysis and esterification, potential added costs due to need of more than 24 h to achieve comparable biodiesel yields and extra post-transesterification refining reactions are among the remaining drawbacks for the environmentally friendlier enzymatic catalysis of crude Jatropha oil to become an economically viable alternative to chemical catalysis.

## Materials and methods

### Vegetable oils

**Fig. 3:**
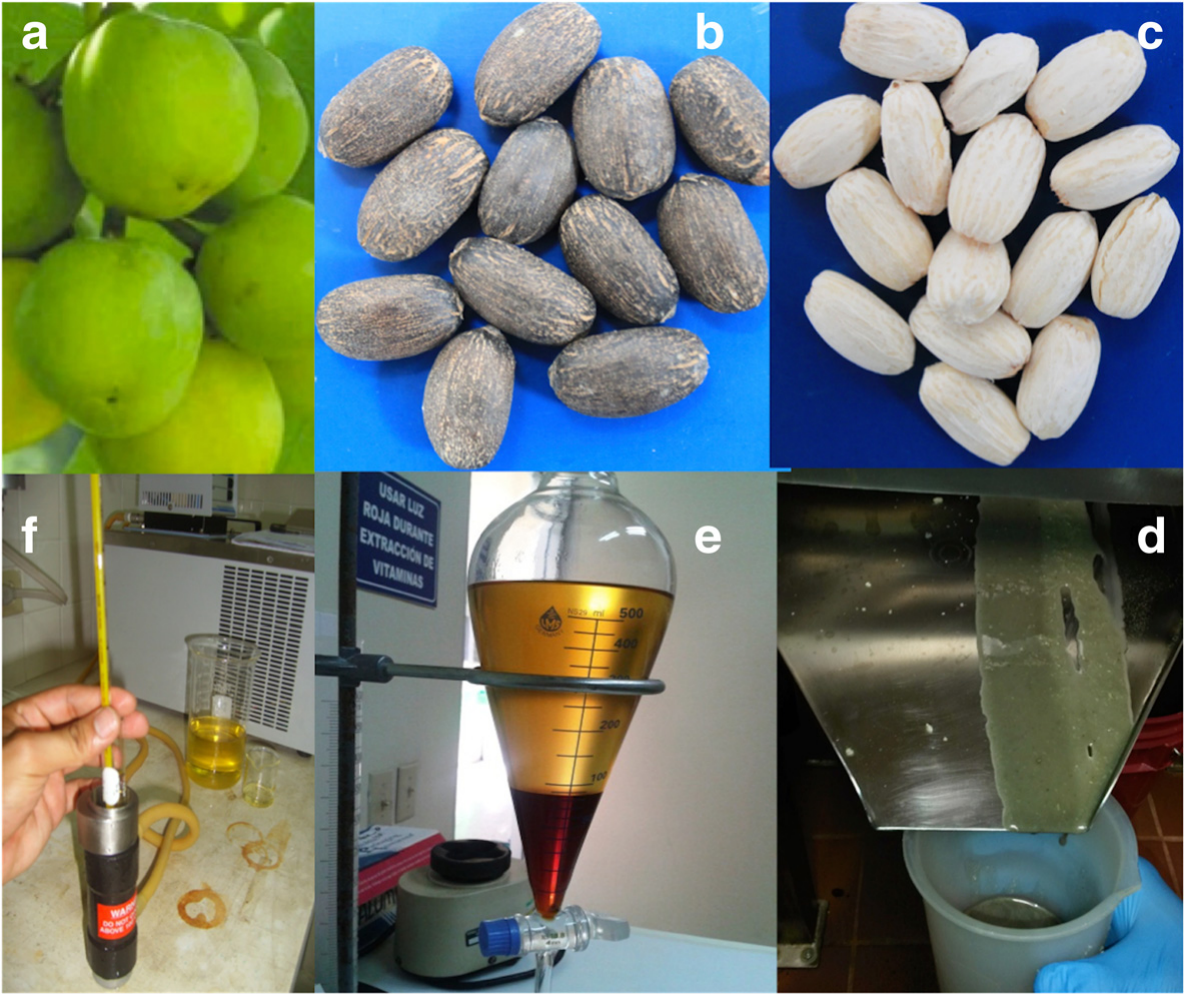
Production of Jatropha biodiesel. **a** Fruits of Jatropha, Cabo Verde variety. **b** Mature seeds of Jatropha. **c** Dehulled seeds of Jatropha. **d** Mechanical Jatropha oil extraction with expeller. **e** Jatropha biodiesel at phase separation step. Top phase is biodiesel and bottom phase is glycerol. **f** Cloud point measurement of Jatropha biodiesel

RBD palm oil was purchased from Corporacion Dinant (Tegucigalpa, Honduras). Jatropha fruits from Cabo verde variety (Fig. 3a) were harvested from the germoplasm collection at EAP Zamorano University (Honduras). Seeds were manually separated from the fruit (Fig. 3b) and hull was removed (Fig. 3c) with a DME-100 dehuller (Ecirtec LTDA. Bauru, SP, Brazil). Jatropha oil was extracted from dehulled seeds with an MPE-40 expeller (Fig. 3d), filtered with an FPE-20 press-filter and degummed in a 25 kg open reactor, all from Ecirtec LTDA. (Bauru, SP, Brazil). FFA content of palm and Jatropha oils was <0.5 %.

### Chemicals and enzymes

An acrylic resin-immobilized non-specific lipase from *Candida antarctica* (10,000 PLU/g) (CA) and a free, soluble, 1,3 specific liquid lipase from *Thermomyces lanuginosus* (100,000 LU/g) (TL) were purchased from Sigma-Aldrich Co. (St. Louis, MO, USA). Isooctane (Uvasol 99.8 %) was obtained from Merck (Darmstadt, Germany), methyl heptadecanoate (GC, >99 %) from Sigma-Aldrich Co. (St. Louis, MO, USA) and FAME standards GLC-463 and GLC-714 were procured from Nu-Chek Prep Inc. (Elysian, MN, USA). Karl Fischer Hydranal composite 5 was purchased from Sigma-Aldrich Co. (St. Louis, MO, USA). All other chemicals were of reagent grade.

### Experimental design

The 2 × 2 factorial experiment was evaluated using a completely randomized design (CRD) with three replicates. Palm and Jatropha oil were transesterified into biodiesel with three catalysts: two enzymes (CA and TL) and one alkali (NaOH, control).

### Transesterification

The transesterification was carried out according to the working conditions described in Table 4. Enzymes working conditions were based on reported optimization studies for CA [1, 26] and TL [3, 10–12].

**Table 4:** Working conditions of catalysts used in transesterification

Catalyst	% Catalyst	% Water	Molar ratio (Methanol:oil)	Temperature (°C)	Time (h)
NaOH	1	0	6:1	60	1
TL	0.75	2	1.5–1	35	24
CA	14	0	3:1	40	24

Data refers to previously optimized working conditions for transesterification of Jatropha and palm oil with alkaline (NaOH) and enzymatic catalysts (TL, CA)

Oil (50 ml) was added in a 250 ml erlenmeyer and stirred with a hot plate (Cimarec Thermo Scientific, Waltham, MA, USA) set at the specified temperature and 200 rpm. Methanol was added stepwise (33 % at reaction time 0 h and 67 % within 1 to 10 h) at the specified molar ratio to treatments with enzymatic catalyst to prevent enzyme inhibition [3]. Water and enzymes were added to corresponding Erlenmeyers with oil and methanol. For control treatments, NaOH and methanol were mixed previously at the indicated amounts (Table 4) and resulting sodium methoxide was added to oil under constant stirring. Reaction time was 24 h.

### Methyl ester separation and drying

Methyl ester phase was separated from glycerol and enzyme phases by centrifugation (Damon/IEC model K115, Thermo Scientific, Waltham, MA, USA) 20 min at 2500 rpm. Top methyl ester phase (Fig. 3e) was extracted, washed twice with 20 ml deionized water at 50 °C and dried 24 h at 105 °C in a convection oven (model 750f, Thermo-Fisher Scientific, Waltham, MA, USA) or until water content dropped below ASTM D6751-11b maximum limit (0.05 % volume). Weight of dry methyl ester phase was recorded (g).

A 10 μl sample of dry methyl esters was mixed with 25 μl of 20 mM heptadecanoic methyl ester (internal standard) and 465 μl of isooctane in an amber vial with 50 mg sodium sulfate for GC analysis [26].

### FAME GC analysis

Samples prepared as described above were analyzed by injecting 1 μl into an Agilent 7890 gas chromatograph, equipped with a SP-2560 capillary column (100 m × 250 μm × 0.25 μm). The column temperature was kept at 180 °C for 1 min, heated to 215 °C at 20 °C/min, and then maintained for 65 min. The temperatures of the injector and detector were set at 260 and 280 °C, respectively. All samples were measured in duplicate. Percent biodiesel yield was defined as fatty acid esters amount produced divided by the initial amount of Jatropha oil (g/100 g).

### Biodiesel quality

Biodiesel quality was compared with ASTM D 6751 standard. FAME was measured by AOCS Ce 2b-11 method by GC-FID with a capillary column (100 m × 250 μm × 0.25 μm) (Fig. 2). Cetane number (CN) was calculated based on FAME profile with Bamgboye and Hansen equation [27]. Percent moisture was measured by AOCS Ca 2e-84 by Karl Fischer titration, oil stability index (OSI) by AOCS Cd 12b-92 and reported in h, and percent free fatty acid (% FFA) by AOCS Ca 5a-40 (titration). Kinematic viscosity (mm^2^/s) was calculated by measuring dynamic viscosity (mPa.s) with a Brookfield rheometer (model LVDV-III Ultra Middleboro, MA, USA) and dividing it by the biodiesel density. Cloud point (°C) was measured according to ASTM D2500 method (Fig. 3f).

### Statistical analysis

Statistical analyses were performed with SAS v. 9.3 (SAS Institute, Cary, NC, USA). A Proc GLM procedure was used for ANOVA, followed by LSD means separation test if no significant interaction between oil and catalyst interaction was found, otherwise a LS Means procedure was employed.

## Abbreviations

TL: *Thermomyces lanuginosus* enzyme
CA: *Candida antarctica* lipase Benzyme
RBD: Refined, bleached and deororized oil
FFA: Free fatty acid
FAME: Fatty acid methyl ester
GC-FID: Gas chromatograph with flame ionization detector
CN: Cetane number
OSI: Oil stability index
LSD: Least significant difference means separation test
SD: Standard deviation
% CV: Percent coefficient of variation
